# Motor Imagery Ability and Motor Imagery Perspective Among Professional Football Players

**DOI:** 10.3390/healthcare13233045

**Published:** 2025-11-25

**Authors:** George Plakoutsis, Eleftherios Paraskevopoulos, Georgios Krekoukias, Anna Christakou, Maria Papandreou

**Affiliations:** 1 Laboratory of Advanced Physiotherapy, Department of Physiotherapy, University of West Attica, 12243 Athens, Greece; 2 School of Physical Education and Sports Science, National and Kapodistrian University of Athens, 17237 Dafne, Greece; 3 Department of Physiotherapy, University of Peloponnese, 23100 Sparta, Greece

**Keywords:** motor imagery, imagery ability, football players, ankle sprains, return-to-play

## Abstract

**Background:** Motor Imagery (MI) refers to the mental simulation of movement without physical execution and activates brain areas involved in motor control. Its use in sports rehabilitation is growing due to its potential to promote recovery, reduce fear of re-injury, and maintain neuromuscular engagement. However, the relationship between MI vividness and preferred imagery perspective remains underexplored in professional athletes. The purpose of this study was to examine the effects of a structured MI intervention on imagery vividness—across internal visual (IVI), external visual (EVI), and kinesthetic (KVI) perspectives—during rehabilitation in professional football players. **Methods:** Fifty-eight professional football players (aged 18–35) recovering from lateral ankle sprains were randomly assigned to an MI group (guided imagery audio) or a Relaxation comparison group (relaxation instructions). Both followed a standardized 4-week physiotherapy program. MI vividness was assessed over six sessions using the Vividness of Movement Imagery Questionnaire-2 (VMIQ-2). **Results:** A mixed ANOVA revealed a significant main effect of time, with both groups showing improved imagery vividness across sessions (reduced VMIQ-2 scores). No significant time × group interaction was observed, indicating similar improvement patterns. Among the three perspectives, IVI showed the most pronounced improvement. **Conclusions:** Repeated engagement with cognitive protocols, even without explicit MI instruction, appears to enhance imagery vividness during rehabilitation. The findings highlight the relevance of internal visual imagery for professional athletes recovering from injury and support the integration of MI-based techniques in physiotherapy programs.

## 1. Introduction

Motor imagery (MI) is a cognitive simulation process in which individuals mentally represent perceptual information without sensory input [1,2]. It involves the mental rehearsal of an action without physical movement [3], activating brain structures similar to those engaged in voluntary movement [4]. According to the motor simulation theory [4,5,6], mentally rehearsing a movement engages overlapping neural networks with actual motor execution, including the supplementary motor area, premotor cortex, and parietal regions [7,8,9]. This shared activation forms the neurophysiological basis for motor imagery and supports its use in injury rehabilitation, where physical movement may be restricted but motor representations remain accessible. As such, MI offers a non-physical method to maintain or even strengthen neural circuits involved in sport-specific actions during periods of physical inactivity [1,7,8,9,10].

MI plays a crucial role in enhancing athletic performance across various sports, particularly in football. Football is the most famous sport globally due to its high-intensity physical demands and risk of injuries, with studies showing that footballers experience a higher rate of injuries compared to many other sports, particularly due to frequent sprinting, tackling, and jumping. Studies show that despite the risk of injury, the appeal of football remains unmatched due to its simplicity, accessibility, and the high level of physical and mental skills required to succeed [8,9]. By vividly visualizing actions such as dribbling, passing, or scoring, football players can strengthen neural pathways associated with movement, improving muscle memory and reaction times [8]. This mental rehearsal helps athletes refine technique, develop strategic awareness, and build confidence under pressure [1,6,8]. Studies show that combining MI with physical training enhances coordination, reduces performance anxiety, and accelerates skill acquisition [8].

Injuries in sports often lead to both physical and psychological distress, with common psychological responses including depression, anger, and anxiety, especially in cases of severe injuries [11]. Research indicates that MI can boost self-efficacy, self-confidence, muscle strength, and overall athletic performance [12,13,14]. Athletes undergoing rehabilitation often experience high stress, which can impede recovery. MI can help mitigate stress, reduce pain and anxiety, and shorten the return-to-play period [15]. By employing MI, athletes can maintain self-confidence and self-motivation throughout rehabilitation, promoting optimal recovery [16,17].

MI ability refers to an individual’s capacity to generate and control vivid mental images and retain them effectively [7,18,19]. Two key attributes of MI ability are vividness and controllability [19]. The efficacy of MI is greater in individuals with higher MI vividness [7], but this ability can improve with practice [20]. Notably, differences in MI vividness exist between athletes in various sports and between those who participate in team versus individual sports [21].

MI can be categorized into visual imagery (VI) and kinesthetic imagery (KI). VI can further be divided into external visual imagery (EVI), where individuals imagine themselves performing an action as an external observer (third-person perspective), and internal visual imagery (IVI) where they visualize the action from their own viewpoint (first-person perspective) [8,19,22,23]. In contrast, KVI involves mentally experiencing the movement with a focus on sensory and proprioceptive feedback [19,23,24]. Understanding these perspectives is essential for optimizing MI application in sports rehabilitation.

Assessing MI is complex, as it is a cognitive process [15] with a multidimensional construct [7]. Various methods, including self-reported questionnaires [19,22], neuroimaging [4,5,6] and responses from the autonomic nervous system at the peripheral level [3,8,10], have been used to evaluate MI vividness. The most commonly employed method is self-reported questionnaires [22,23], with the Vividness of Movement Imagery Questionnaire-2 (VMIQ-2) being the most widely used in sports research [8,9,19,22,24]. The VMIQ-2 is a validated tool for assessing MI vividness, including vividness and controllability [19,22], and is often used by sports physiotherapists and psychologists to evaluate athletes’ MI capacity [8,9,25,26].

While MI has been extensively investigated in the context of skill acquisition and performance enhancement, its application during the rehabilitation process remains underexplored. Few studies have examined how imagery vividness and perspective evolve during recovery from injury, particularly in professional football populations. Based on the existing gap in the literature, the present study aimed to investigate whether the vividness of motor imagery improves over time during rehabilitation in professional football players. The research questions guiding this study were: 1. Does motor imagery vividness change across six sessions during a 4-week rehabilitation program? 2. Do different imagery perspectives (IVI, EVI, KVI) and type of intervention (MI vs. Relaxation) influence vividness over time?

We hypothesized that MI vividness would improve over time across all participants. Additionally, we explored whether the imagery perspective (IVI, EVI, KVI) and intervention type (MI vs. Relaxation) would influence the pattern of change.

## 2. Materials and Methods

### 2.1. Participants

The study involved 69 professional football players selected through stratified sampling. After an initial assessment, 5 participants chose to withdraw, and 3 were disqualified due to functional ankle instability, leaving a final cohort of 58 athletes aged 18–35 years. These participants were randomly assigned to 2 groups using a closed-envelope method: the MI group (n = 29) and the Relaxation comparison group (n = 29), (Table 1).

To qualify for the study, participants needed a Grade II lateral ankle sprain diagnosis from a sports medicine doctor with at least 5 years of experience and had to be in the return-to-play rehabilitation phase. The sample included players across all field positions, with the majority being midfielders and defenders, although positional breakdowns were not used in statistical analysis. Players were active members of their respective teams, with an average weekly playing time exceeding 45 min prior to injury. Exclusion criteria encompassed visual, vestibular, or neurological impairments, recent lower limb fractures or musculotendinous injuries, functional ankle instability [27], recent surgeries, or a history of concussion.

All participants were professional football players from Greece’s Super League 1, Super League 2, or the Football League. The research was conducted at the Laboratory of Advanced Physiotherapy (LadPhys) at the University of West Attica. Ethical approval was granted by the University of West Attica’s Ethics Committee (Approval No18030), adhering to the Declaration of Helsinki, and all athletes provided written informed consent.

### 2.2. Procedures

This randomized controlled trial was designed to assess MI vividness and perspective changes in professional football players undergoing rehabilitation. All participants followed a 4-week standardized physiotherapy program that incorporated balance training as part of the return-to-play phase. Sessions were conducted twice weekly (total of 6 sessions), with each session including warm-up, balance exercises, and cool-down phases. To ensure consistency across participants, no independent mental imagery practice was permitted between sessions. Participants were explicitly instructed not to perform MI exercises outside the supervised intervention setting and were regularly reminded of this restriction.

Prior to initiating the intervention, a licensed physiotherapist with 5 years of clinical experience provided a briefing and demonstrated the exercise protocol to ensure consistency in execution. Anthropometric measurements (height and body mass) were recorded using calibrated instruments (SECA stadiometer and Xiaomi Mi Body Scale 2) [28]. The duration of the MI protocol was set at 4 weeks to correspond with the final stage of rehabilitation, during which athletes typically transition from clinical settings to sport-specific training. This period is considered optimal for integrating cognitive strategies that support neuromuscular reconditioning. Furthermore, previous studies have demonstrated that interventions lasting 3 to 5 weeks are sufficient to produce measurable improvements in imagery vividness and motor performance in athletes [8,26].

### 2.3. Main Outcome Measures

#### 2.3.1. Vividness of Movement Imagery Questionnaire-2

The VMIQ-2 is a tool designed to assess the vividness of MI using twelve specific movement scenarios. Each scenario is evaluated on a 5-point Likert scale, where 1 represents a perfectly clear and vivid image, and 5 indicates no image at all. The questionnaire explores 3 distinct visual imagery perspectives: external visual imagery (EVI), internal visual imagery (IVI), and kinesthetic visual imagery (KVI). Participants are instructed to visualize themselves performing the twelve scenarios from each of these perspectives [22].

The scenarios included in the VMIQ-2 are as follows: 1. Walking, 2. Running, 3. Kicking a stone, 4. Bending down to pick up a coin, 5. Running upstairs, 6. Jumping sideways, 7. Throwing a stone into water, 8. Kicking a ball into the air, 9. Running downhill, 10. Riding a bike, 11. Swinging on a rope, and 12. Jumping off a high wall. The scoring system interprets lower scores (VMIQ-2 score < 26) as indicative of high imagery ability, while higher scores (VMIQ-2 score > 36) suggest lower imagery ability.

The Greek version of the VMIQ-2, known as VMIQ-2-GR, has demonstrated strong validity and reliability, with an intraclass correlation coefficient (ICC) exceeding 0.92 [22].

#### 2.3.2. Intervention Protocol

Both the MI and Relaxation groups completed the same physical rehabilitation program, consisting of neuromuscular balance exercises structured into 3 segments:

- Warm-up (8 min): light running, dynamic stretching, and bodyweight drills (e.g., squats, knee lifts),
- Balance Training (15 min): progressive static and dynamic tasks on unstable surfaces, including single-leg stance on foam and wobble boards, dynamic transitions, and controlled squats,
- Cool-down (7 min): static lower limb stretches targeting major muscle groups [29,30,31,32].

After the balance component, participants in the MI group received a 20-min guided motor imagery session. The protocol included an initial relaxation phase followed by mental rehearsal of the balance tasks. All instructions were delivered via pre-recorded audio by a certified MI instructor, ensuring consistency. The recordings were produced using WaveLab version 9 and Cubase version 10, with a condenser microphone (NT1, 4th generation, Rode, Sydney, Australia). Playback was conducted using a MacBook Pro (M1, Apple Inc., Cupertino, CA, USA) and standardized headphones (Keiji, HD-2400G, Zeroground, Athens, Greece). Sessions were conducted in a quiet, dimly lit room with standardized headphones.

In contrast, the Relaxation comparison group listened to general relaxation scripts without any movement-related content. Both groups completed the VMIQ-2-GR questionnaire before each session to assess imagery vividness across internal, external, and kinesthetic dimensions.

#### 2.3.3. Statistical Analysis

The data were summarized using descriptive statistics, with continuous variables expressed as means and standard deviations, and categorical variables presented as counts and proportions. Differences in the anthropometric characteristics of the athlete population were evaluated using the chi-square trend test and chi-square test [33], as detailed in Table 1. The Kolmogorov–Smirnov test was applied to assess the normality of the data distribution.

To examine changes over the 4-week period-6 MI sessions (the ‘time’ factor) and differences between the 2 groups, a mixed ANOVA was conducted. This analysis assessed the main effects of Time (within-subjects factor) and Group (between-subjects factor), as well as their interaction [34]. The clinical significance of the results was evaluated using partial eta squared (*η*^2^) to measure effect size. Effect sizes were categorized as small (*η*^2^ ≥ 0.01), medium (*η*^2^ ≥ 0.06), or large (*η*^2^ ≥ 0.14) [35,36]. No participants dropped out of the study, and no missing data were observed across the 6 assessment time points. All statistical analyses were performed using SPSS version 26 (Statistical Package for the Social Sciences, SPSS Inc., Chicago, IL, USA).

## 3. Results

### Vividness of Movement Imagery Questionnaire-2 (VMIQ-2-GR)

A mixed ANOVA was conducted to examine the effects of Time (within-subjects factor: six MI sessions) and Group (between-subjects factor: MI intervention vs. Relaxation) on imagery vividness scores for the three modalities: external visual imagery (EVI), internal visual imagery (IVI), and kinesthetic visual imagery (KVI). Since Mauchly’s Test of Sphericity indicated a violation of the sphericity assumption, Greenhouse-Geisser and Huynh-Feldt corrections were applied (Table 2).

A significant main effect of Time was found for all imagery modalities, indicating that vividness scores changed significantly over the six sessions. The effect sizes were large (EVI: *η*^2^ = 0.568, IVI: *η*^2^ = 0.540, KVI: *η*^2^ = 0.402), demonstrating that time-related factors contributed substantially to the variance in imagery vividness. These effects remained significant after applying the necessary corrections.

A statistically significant Time × Group interaction emerged only for the IVI modality (*η*^2^ = 0.215, *p* = 0.024), suggesting that the MI intervention group showed greater improvement in internal visual imagery compared to the Relaxation comparison group. No significant interaction was observed for EVI or KVI, indicating that both groups followed a similar pattern of change over time for those modalities. There was also no main effect of Group in any of the three modalities.

Pairwise comparisons between sessions revealed significant decreases in vividness scores from early to later sessions, particularly between Session 1 and Session 6, and between Sessions 2 and 6. This suggests a general trend of vividness improvement through repeated engagement, regardless of group assignment, though IVI showed a group-specific enhancement (Table 3).

## 4. Discussion

This study explored how imagery vividness evolved during rehabilitation in professional football players, with a focus on preferred imagery perspectives across six motor imagery sessions. The findings revealed a significant main effect of Time, indicating that MI vividness, as measured by EVI, IVI, and KVI scores, improved significantly across the six MI sessions. This improvement was consistent across both the MI intervention and Relaxation comparison groups, as evidenced by the lack of a significant Time × Group interaction. These results suggest that repeated MI practice, regardless of the specific intervention, plays a critical role in enhancing MI vividness over time. However, the absence of significant between-group differences highlights that the MI intervention did not produce differential effects compared to Relaxation, at least within the context of this study.

The significant reduction in IVI scores over the six sessions suggests that the IVI perspective may be particularly effective for professional football players during rehabilitation. This aligns with previous research by Hardy and Callow [37], who found that IVI was more effective than EVI for enhancing performance in experienced athletes. Similarly, Olsson et al. [26] demonstrated that IVI improved sports performance more effectively than conventional physical training programs in professional high jumpers. The preference for IVI in this study may reflect the athletes’ familiarity with internal perspectives, which closely mirror real-life movement execution and are controlled by visual and visuo-spatial processes in the occipital cortex and superior parietal lobe [37].

The lack of a significant Time × Group interaction suggests that both the MI and Relaxation comparison groups experienced similar improvements in MI vividness over time. This finding raises important questions about the mechanisms underlying MI training. It is possible that the act of engaging in regular MI practice, rather than the specific content of the intervention, drives improvements in MI vividness. This is consistent with the work of Callow and Roberts [24], who found that MI perspectives are often experienced concurrently rather than sequentially. The observed improvements in both EVI and IVI perspectives further support the idea that professional athletes may utilize MI perspectives differently depending on the task and context [38,39,40,41].

Several factors could influence the preference of MI perspective (EVI, IVI, KVI), used in MI sessions [6]. A successful movement requires a convey of all perceived information into action and the athlete must consider the dynamics of the environment and his/her biomechanical limits [39]. Furthermore, MI perspective should be regulated by the nature of the task (e.g., sports performance, rehabilitation), environment (e.g., sports field, rehabilitation center) and individual characteristics (e.g., athlete) [38,39,40,41]. Taking into consideration that the perspectives of imagery could not be separated, the application of both IVI and KVI appears reasonable and achievable.

It is possible that the act of engaging in regular MI practice, rather than the specific content of the intervention, drives improvements in MI vividness. Although no significant differences were observed between the MI and Relaxation comparison groups, it is important to consider that both interventions involved structured, guided cognitive activity. The relaxation protocol itself constitutes an active psychological stimulus known to influence attentional focus and anxiety—factors that may indirectly enhance imagery vividness [7,8,9]. Therefore, the absence of group differences should not be interpreted as indicating that MI has no specific effects, but rather that both conditions may share common cognitive mechanisms, such as increased self-awareness, expectancy, and mental engagement [10]. Future studies should consider including a passive control group receiving no cognitive intervention or an unrelated audio stimulus to better isolate MI-specific contributions.

Beyond the need for improved control conditions, it is equally important to explore the potential mechanisms that may explain the observed improvements in imagery vividness across time. The observed improvement in imagery vividness across sessions, despite the absence of group differences, may reflect non-specific mechanisms rather than MI-specific training effects. Repeated administration of the VMIQ-2 may have enhanced participants’ familiarity with introspective ratings, leading to more refined self-assessments over time. Additionally, both interventions—motor imagery and relaxation—may have engaged overlapping cognitive processes such as attentional control, expectancy modulation, and metacognitive awareness [7,15]. These mechanisms are supported by neurocognitive models indicating that structured mental practice can recalibrate proprioceptive expectations and activate motor simulation networks, even in the absence of overt movement [15]. The potential contribution of generalized cognitive engagement to vividness gains should therefore be acknowledged alongside any MI-specific interpretations.

Furthermore, Guillot et al. [42] in their study reported that MI is a mental process and IVI perspective relies on visual and visuo-spatial processes which are controlled by the occipital cortex and the superior parietal lobe [42]. On the contrary, Jackson, Meltzoff and Decety [43], in their study reported that the EVI perspective requires additional visuo-spatial transformation and it was controlled by the lingual gyrus [43]. Thus, the use of EVI perspective require athletes to go through a more difficult mental process in comparison with the IVI perspective. Hua et al. [23], in their study support the view that EVI perspective is more useful when learning a new motor skill whereas IVI perspective is more useful for the development of strategies regardless the time of sport [23].

Many studies have shifted their interest on the MI vividness, the type of sport and the level of experience [23,44,45]. In the studies of Arvinen-Barrow et al. [44] and Watt et al. [45], the findings revealed that professional athletes tended to adopt MI techniques with a high frequency compared to the amateur athletes. Also, athletes participated in sports such as rugby and martial arts had a higher level of MI engagement compared to athletes who participated in sports such as golf [44]. These findings are in accordance with the findings of our study where professional football players showed statistically significant improvement of MI vividness in both groups 4 weeks after intervention.

Consequently, the results of this study highlighted the strong relationship between MI vividness and IVI perspective preference in professional football players. These findings align with previous research suggesting that the IVI perspective is particularly beneficial for experienced athletes [26,37]. The structured cognitive interventions, including MI and relaxation-based protocols, may support imagery vividness during rehabilitation in professional football players. A key consideration is the cognitive demand associated with different MI perspectives. The IVI perspective, which closely mirrors real-life movement execution, may facilitate a stronger neural activation pattern [42]. The EVI perspective, in contrast, may be more useful for analyzing technique and refining motor skills from an external viewpoint [43]. Additionally, the KVI perspective, which engages proprioceptive feedback, may be beneficial for athletes recovering from injuries where sensory reintegration is critical [40].

### 4.1. Clinical Implications

The findings of this study have significant clinical implications for sports rehabilitation and performance enhancement. The significant main effect of Time underscores the importance of repeated MI practice in improving MI vividness, regardless of the specific intervention. This suggests that MI can serve as an effective adjunct to conventional rehabilitation programs, allowing athletes to engage in cognitive practice when physical movement is restricted. By integrating MI into rehabilitation protocols, clinicians can help athletes maintain neuromuscular activation and mental readiness, reducing the risk of performance deterioration during injury recovery.

One of the key benefits of MI in rehabilitation is its ability to address psychological barriers to recovery, such as fear of re-injury. MI has been shown to promote confidence and psychological resilience, helping athletes visualize successful movement execution and rebuild trust in their physical abilities [10]. This mental preparation can lead to increased adherence to rehabilitation protocols and improved return-to-play outcomes [9].

Moreover, MI offers an accessible and cost-effective rehabilitation tool that requires no specialized equipment. It can be implemented in various settings, including clinical environments, sports facilities, and home-based rehabilitation programs. Clinicians can personalize MI protocols to match the specific needs and preferences of each athlete, ensuring optimal engagement and effectiveness [46].

MI also has applications beyond injury recovery, playing a crucial role in injury prevention and performance optimization. Athletes who regularly incorporate MI into their training routines can enhance motor learning, refine movement patterns, and develop a heightened awareness of body mechanics [6]. This proactive approach can reduce the likelihood of future injuries by reinforcing proper movement execution and promoting efficient neuromuscular control [10,47].

Another significant clinical implication of this study is the potential for MI to be integrated with other psychological techniques, such as mindfulness and cognitive-behavioral strategies. Combining MI with relaxation techniques and goal-setting strategies may further enhance its effectiveness in reducing anxiety, improving focus, and maintaining motivation throughout rehabilitation and training [48,49].

Furthermore, MI can be particularly beneficial for athletes recovering from long-term injuries or surgical interventions. Traditional rehabilitation exercises may be limited by physical constraints, but MI allows athletes to continue mental engagement with their sport [10]. Studies have demonstrated that MI can help maintain muscle activation patterns and motor planning capabilities, reducing the impact of prolonged immobilization and facilitating a smoother transition back to full activity [50].

Future research should explore the long-term effects of MI training on injury prevention and recovery, as well as its potential applications across different sports and levels of competition. Investigating the impact of MI on youth athletes and amateur sports participants could provide valuable insights into its broader benefits. Additionally, advancements in neuroimaging and physiological monitoring could enhance our understanding of MI’s underlying mechanisms, leading to more refined and targeted interventions.

### 4.2. Limitations

This study presents several limitations that should be acknowledged. First, the repeated administration of the VMIQ-2 across six sessions may have introduced measurement reactivity, as participants may have become progressively more familiar with the structure and content of the scale. This could have influenced their self-perception or response style, leading to artificially inflated vividness scores over time. Also, the relatively short duration of the intervention (six sessions over 4 weeks) limits the strength and generalizability of the findings. While improvements in imagery vividness were observed, these changes likely reflect short-term adaptations such as increased task familiarity or attentional engagement. The current results should therefore be interpreted as preliminary, and not indicative of long-term neuroplastic changes.

Second, due to the nature of the interventions, athletes could not be blinded to their assigned group, and expectancy effects were neither measured nor controlled. These factors may have contributed to non-specific improvements in self-reported imagery vividness.

Third, the study did not include any behavioral or physiological outcome measures (e.g., pain levels, balance performance, proprioceptive sensitivity, or time to return-to-play). As such, while the findings suggest enhanced subjective vividness, no conclusions can be drawn regarding the actual functional impact of the interventions.

Future research should aim to combine self-report measures with objective indicators of functional recovery, and incorporate passive control conditions to isolate the specific contributions of ΜΙ from general cognitive engagement. Studies with longer durations and neurophysiological assessments are needed to clarify the underlying mechanisms and the sustainability of such effects.

## 5. Conclusions

This study examined how MI vividness and perspective evolved during rehabilitation among professional football players. The findings demonstrated that imagery vividness improved significantly over the six sessions for both groups, regardless of whether athletes received guided motor imagery or relaxation. Internal visual imagery (IVI) emerged as the most responsive modality, suggesting a natural preference or enhanced effectiveness of first-person perspective imagery in this population.

These results support the integration of MI strategies—regardless of delivery mode—into injury rehabilitation protocols to reinforce mental rehearsal and psychological readiness. The emphasis on internal perspective imagery may further enhance ecological validity when applying MI to professional athletes.

Further studies may explore the mechanisms driving perspective preferences and extend these findings across other sports and recovery timelines.

## Figures and Tables

**Table 1:** Demographic and injury-related characteristics of the athletes.

Variable	Total (N = 58)	MI Group (n = 29)	Relaxation Group (n = 29)	Statistical Test	*p*-Value
Age (years) (M ± SD)	20.5 ± 3.3	20.5 ± 3.3	21.2 ± 3.1	Independent *t*-test	0.37 (NS)
BMI (kg/m^2^) (M ± SD)	22.3 ± 1.9	22.8 ± 1.7	21.8 ± 2.1	Independent *t*-test	0.05
Years of Training (M ± SD)	11.1 ± 2.7	11.0 ± 2.8	11.2 ± 2.6	Independent *t*-test	0.81 (NS)
Training Hours per Week	12.1 ± 1.5	11.9 ± 1.6	12.3 ± 1.4	Independent *t*-test	0.26 (NS)
Dominant Leg—Right (n, %)	46 (79.3%)	25 (86.2%)	21 (72.4%)	Chi-square	0.19 (NS)
Dominant Leg—Left (n, %)	12 (20.7%)	4 (13.8%)	8 (27.6%)		
Injured Leg—Right	39 (67.2%)	21 (72.4%)	18 (62.1%)	Chi-square	0.40 (NS)
Injured Leg—Left	19 (32.8%)	8 (27.6%)	11 (37.9%)		
Previous LAS (Right)	38 (65.5%)	17 (58.6%)	21 (72.4%)	Independent *t*-test	0.30 (NS)
Previous LAS (Left)	12 (20.7%)	6 (20.7%)	6 (20.7%)		
Previous LAS (Both)	8 (13.8%)	6 (20.7%)	2 (6.9%)		
Total Previous LAS (n, %)				Chi-square for trend	0.35 (NS)
1	30 (51.7%)	17 (58.6%)	13 (44.8%)		
2	21 (36.2%)	9 (31.0%)	12 (41.4%)		
≥3	7 (12.1%)	3 (10.3%)	4 (13.8%)		

NS = no significance, M = Mean, SD = Standard Deviation.

**Table 2:** Mixed ANOVA with Sphericity Corrections revealed significant main effect of Time, indicating that the MI ability changes significantly across the six MI sessions.

Effect	Test	F-Value	Degrees of Freedom (df)	*p*-Value	Partial *η*^2^ (Effect Size)	Interpretation
**Within-Subjects Effects**						
Time	Sphericity Assumed	35.343	5; 280	<0.001	0.387	Significant main effect of Time. Scores change significantly across time points.
	Greenhouse-Geisser	35.343	2.150; 120.413	<0.001	0.387	Significant after Greenhouse-Geisser correction.
	Huynh-Feldt	35.343	2.279; 127.614	<0.001	0.387	Significant after Huynh-Feldt correction.
Time × Group	Sphericity Assumed	0.565	5; 280	0.727	0.010	No significant interaction. Time effects do not differ between groups.
	Greenhouse-Geisser	0.565	2.150; 120.413	0.582	0.010	No significant interaction after Greenhouse-Geisser correction.
	Huynh-Feldt	0.565	2.279; 127.614	0.592	0.010	No significant interaction after Huynh-Feldt correction.
**Between-Subjects Effects**						
Group	-	1.679	1.56	0.200	0.029	No significant differences between MI and Placebo groups overall.
**Sphericity Tests**						
Mauchly’s W	-	0.032	14	<0.001	-	Sphericity violated. Greenhouse-Geisser & Huynh-Feldt corrections were used.
**Pairwise Comparisons—6 sessions (Time)**						
Session 1 vs. Session 3	-	-	-	<0.001	-	Significant difference (*p* < 0.001).
Session 1 vs. Session 6	-	-	-	<0.001	-	Significant difference (*p* < 0.001).
Session 2 vs. Session 6	-	-	-	<0.001	-	Significant difference (*p* < 0.001).
**Estimated Marginal Means—6 sessions (Time)**						
Session 1	-	29.293	-	-	-	Mean score at Time 1 (95% CI: 26.782, 31.804).
Session 6	-	21.569	-	-	-	Mean score at Time 6 (95% CI: 19.620, 23.518).

CI: Confidence Interval.

**Table 3:** Comparison of Mean Imagery Vividness Scores per Session Between the MI and Relaxation Groups (IVI, EVI, KVI Perspectives).

Sessions	IVI (MI)—Means	IVI (Relaxation)—Means	EVI (MI)—Means	EVI (Relaxation)—Means	KVI (MI)—Means	KVI (Relaxation)—Means
1	20.86	23.21	28.07	30.52	23.34	24.83
2	21.59	22.72	26.34	30.07	21.52	23.41
3	20.86	20.00	24.24	26.14	20.76	22.97
4	19.41	18.72	23.28	25.14	19.24	21.90
5	17.97	17.17	21.76	23.59	18.24	21.83
6	16.17	16.69	20.07	23.07	17.28	20.38

EVI: external Visual Imagery; IVI: Internal Visual Imagery; KVI: Kinesthetic Visual Imagery.

## Data Availability

The data presented in this study are available on request from the corresponding author due to ethical and privacy restrictions. The data contain sensitive personal information collected under informed consent, and public sharing is not permitted by the ethics committee. The raw Excel dataset can be provided upon reasonable request.
